# Control of Angiogenesis by Galectins Involves the Release of Platelet-Derived Proangiogenic Factors

**DOI:** 10.1371/journal.pone.0096402

**Published:** 2014-04-30

**Authors:** Julia Etulain, Soledad Negrotto, María Virginia Tribulatti, Diego Omar Croci, Julieta Carabelli, Oscar Campetella, Gabriel Adrián Rabinovich, Mirta Schattner

**Affiliations:** 1 Laboratory of Experimental Thrombosis, Institute of Experimental Medicine, CONICET-National Academy of Medicine, Buenos Aires, Argentina; 2 Institute of Biotechnological Investigations, Technologic Institute of Chascomús-National University of San Martín, CONICET, Buenos Aires, Argentina; 3 Laboratory of Immunopathology, Institute of Biology and Experimental Medicine (IBYME), CONICET, Buenos Aires, Argentina; 4 Laboratory of Functional Glycomics, Faculty of Exact and Natural Sciences, University of Buenos Aires, Buenos Aires, Argentina; University of Torino, Italy

## Abstract

Platelets contribute to vessel formation through the release of angiogenesis-modulating factors stored in their α-granules. Galectins, a family of lectins that bind β-galactoside residues, are up-regulated in inflammatory and cancerous tissues, trigger platelet activation and mediate vascularization processes. Here we aimed to elucidate whether the release of platelet-derived proangiogenic molecules could represent an alternative mechanism through which galectins promote neovascularization. We show that different members of the galectin family can selectively regulate the release of angiogenic molecules by human platelets. Whereas Galectin (Gal)-1, -3, and -8 triggered vascular endothelial growth factor (VEGF) release, only Gal-8 induced endostatin secretion. Release of VEGF induced by Gal-8 was partially prevented by COX-1, PKC, p38 and Src kinases inhibitors, whereas Gal-1-induced VEGF secretion was inhibited by PKC and ERK blockade, and Gal-3 triggered VEGF release selectively through a PKC-dependent pathway. Regarding endostatin, Gal-8 failed to stimulate its release in the presence of PKC, Src and ERK inhibitors, whereas aspirin or p38 inhibitor had no effect on endostatin release. Despite VEGF or endostatin secretion, platelet releasates generated by stimulation with each galectin stimulated angiogenic responses *in vitro* including endothelial cell proliferation and tubulogenesis. The platelet angiogenic activity was independent of VEGF and was attributed to the concerted action of other proangiogenic molecules distinctly released by each galectin. Thus, secretion of platelet-derived angiogenic molecules may represent an alternative mechanism by which galectins promote angiogenic responses and its selective blockade may lead to the development of therapeutic strategies for angiogenesis-related diseases.

## Introduction

In the normal adult, angiogenesis, defined as the growth of new blood vessels from preexisting capillaries, is mostly limited to wound healing, pregnancy, and uterine cycling. This process is regulated by a continuous interplay of stimulators and inhibitors that tightly control the normally quiescent capillary vasculature, and their imbalance contributes to numerous malignant, ischemic, and immune disorders [1].

Emerging evidence implicates a key role for platelets in site-specific neovascularization at ischemic tissues and the tumor microenvironment as they are major storage and delivery vehicles for pro- and antiangiogenic growth factors including vascular endothelial growth factor (VEGF), endostatin, and thrombospondin-1 (TSP-1), and cytokines and chemokines, such as stromal-derived factor 1 α (SDF-1α) and IL-8 among others. These proteins are stored together with coagulation factors in platelet α-granules, which upon activation can degranulate and influence the local angiogenic response [2], [3]. The recent demonstration of separate packaging of these proteins into morphologically distinct populations of α-granules in megakaryocytes and platelets [4], [5], [6], and their differential release based on selective engagement of platelet receptors provides mechanism by which platelets can locally and sequentially modulate angiogenesis [7], [8], [9], [10].

Galectins constitute a family of animal lectins that bind β-galactoside residues through their carbohydrate recognition domains (CRD). According to their structure, galectins are classified into three groups: I) “proto-type” galectins, containing a single CRD including galectin (Gal) -1, -2, -7, -10, -13, and -14, II) chimera-type galectins which contain a single CRD and a large amino-terminal non-lectin domain with Gal-3 being its only representative, and III) tandem-repeat type galectins which have two CRD linked by a peptide sequence of variable length and include Gal-4, -8, -9, and -12 [11]. Galectins are expressed in a wide variety of cells and tissues and play an important role in cellular mechanisms including cell signaling, proliferation, migration, apoptosis, and mRNA splicing [12]. Galectins are also associated with different pathologies such as allergies, autoimmune diseases, atherosclerosis, infectious processes, and cancer [13], [14], [15], [16]. In fact, most of these lectins are up-regulated in several types of tumors and this feature correlates with tumor progression, aggressiveness and acquisition of a metastatic phenotype [16], [17], [18]. In addition, increasing evidence indicates that these proteins are also involved in the pathogenesis of cardiovascular diseases [13]. Recent work from our laboratory has shown that both soluble and immobilized Gal-1 and Gal-8 bind and activate platelets, promoting adhesion, aggregation and granule secretion [19], [20], [21], [22].

A growing body of experimental evidence suggest that galectins, mainly Gal-1, -3 and -8, are directly involved in angiogenesis as they induce endothelial cell proliferation, chemotaxis, *in vitro* capillary tube formation, and *in vivo* neovascularization [23], [24], [25], [26]. Particularly, the Gal-1-N-glycan axis can link tumor hypoxia to vascularization [26] and promotes VEGF-like signals in tumors that are refractory to anti-VEGF therapy [27].

As platelet activation is stimulated by structurally different galectins, in this study we aimed to elucidate whether the release of platelet-derived proangiogenic molecules represents an additional mechanism by which these lectins promote neovascularization. We found that while all three galectins triggered VEGF release, only Gal-8 induced endostatin secretion. However, despite selective release of platelet-derived angiogenic molecules, platelets stimulated by Gal-1, -3 and -8 promoted angiogenic functional responses. Thus, secretion of platelet-derived angiogenic molecules represents an alternative mechanism underlying the proangiogenic activity of galectins.

## Materials and Methods

### Reagents

Endostatin and VEGF ELISA kits, and Human angiogenesis Antibody Array G Series 1000 were from Raybiotech, Inc. (Norcross, GA, USA). Gö-6983, PP1, SB-203580 and U-0126 were from Enzo Life Sciences International, Inc. (San Diego, CA, USA). Recombinant human Gal-1 was produced essentially as described [28]. The recombinant protein was purified by affinity chromatography on an asialofetuin-agarose column and stored at −20°C. Lipopolysaccharide content of the purified samples was tested using a Gel Clot Limulus Test (Cape Cod., East Falmouth, MA, USA). Human Gal-3-coding DNA sequence was modified to improve its expression in bacteria and a His tag was included to favor purification. The sequence was submitted for synthesis (GeneScript) and the resulting gene subcloned into pTrcHisB (Invitrogen). Recombinant human Gal-8 (M isoform) was obtained as described previously [20]. Recombinant human Gal-3 and mouse gal-1-8-1 chimera were produced as described previously [29]. Growth factor-reduced Matrigel was obtained from Becton Dickinson Biosciences (Bedford, MA, USA). MTT [3-(4,5-dimethylthiaxol-2-yl)-2,5-diphaenyltetrazolium bromide] was from Promega Corporation (Madison, WI, USA). Recombinant VEGF-A, goat anti human VEGFR2/KDR/Flk-1, mouse anti human VEGF-A neutralizing antibody, and the corresponding irrelevant IgGs were purchased from R&D Systems (Minneapolis, MN, USA). Rabbit anti-human EGF neutralizing antibody and control IgG were obtained from Cell Signaling Technology (Danvers, MA, USA).

### Preparation of Human Platelets

Blood samples were obtained from healthy donors who had not taken nonsteroidal antiinflammatory drugs in the 10 days before sampling. This study was conducted according to the principles of the Declaration of Helsinki. The study was approved by the Institutional Review Board of the National Academy of Medicine, Buenos Aires, Argentina. All patients provided written informed consent for the collection of samples and subsequent analysis. Blood was drawn directly into plastic tubes containing sodium citrate (3.8%). Platelet rich plasma (PRP) was obtained by centrifugation of the blood samples (180×*g* for 10 min). For washed platelet (WP) suspensions, PRP was centrifuged in the presence of prostacyclin (PGI_2_) (75 nM) (890×*g* for 10 min), and the platelets were washed in washing buffer (140 mM NaCl, 10 mM NaHCO_3_, 2.5 mM KCl, 0.5 mM Na_2_HPO_4_, 1 mM MgCl_2_, 22 mM sodium citrate, 0.55 mM glucose, 0.35% BSA, pH 6.5). Finally, platelets were resuspended in Tyrode’s buffer (134 mM NaCl, 12 mM NaHCO_3_, 2.9 mM KCl, 0.34 mM Na_2_HPO_4_, 1 mM MgCl_2_, 10 mM HEPES, 5 mM glucose, 0.35% BSA, pH 7.4) at a concentration of 4×10^8^/ml, unless otherwise stated. CaCl_2_ (1 mM) was added 1 min before platelet stimulation.

### ELISA Assays for VEGF and Endostatin Release

Briefly, WPs (500 µl at 4×10^8^/ml) were stimulated with Gal-1, -3 or -8 for 5 min. Cell suspensions were centrifuged twice (first at 1100×*g* for 5 min and then at 9300×*g* for 5 min) in the presence of PGI_2_ (75 nM) and supernatants were stored at −80°C until assayed. In some experiments, WPs were incubated for 30 min before stimulation with aspirin (ASA, 500 µM), or with specific inhibitors of PKC (Gö-6983, 1 µM), Src (PP1, 5 µM), p38 (SB-203580, 25 µM) and pERK (U-0126, 10 µM). The concentrations of the inhibitors were selected from pilot studies and were the minimal that completely suppressed phosphorylation of the specific target proteins as previously described [30]. The concentration of ASA used was the minimal that abrogated arachidonic acid-induced platelet aggregation. To obtain platelet lysates, platelet pellets were suspended with distilled in H_2_O in presence of Triton X100 (0.1%) and cells were hydrolyzed by sonication. Ionic concentration was restored by addition of Tyrode’s buffer (10X) and supernatants were obtained. Release of VEGF and endostatin was measured by using commercial ELISA kits according to the manufacturer’s instructions.

### Human Angiogenic Factors Array

WPs (500 µl at 4×10^8^/ml) were stimulated with Gal-1, -3 or -8 for 5 min. Cell suspensions were centrifuged twice (first at 1100×*g* for 5 min and then at 9300×*g* for 5 min) in the presence of PGI_2_ (75 nM) and supernatants were stored at −80°C until assayed. Release of angiogenic factors was measured by using commercial array kits according to the manufacturer’s instructions.

### Endothelial Cell Culture

Human microvascular endothelial cells 1 (HMEC-1) were obtained from the Center of Disease Control and Prevention (CDC, Atlanta, USA). Cells were grown in RPMI supplemented with fetal bovine serum (10%), L-glutamine (2 mM) and streptomycin (100 µg/ml), and penicillin (100 U/ml) at 37°C in a humidified 5% CO_2_ incubator. In selected experiments VEGFR2/KDR was blocked with an anti-VEGFR2 neutralizing monoclonal antibody. Some experiments were also performed in the presence of VEGF-A or EGF receptor (EGFR) neutralizing antibodies.

### Endothelial Cell Proliferation

Cell proliferation was determined by using the MTT [3-(4,5-dimethylthiaxol-2-yl)-2,5-diphaenyltetrazolium bromide] assay. Briefly, HMEC-1 (250 µl at 2.5×10^4^/well) were incubated in 48-well plates with sucrose (50 mM) or lactose (50 mM) for 30 min in the presence or absence of the VEGFR2 neutralizing antibody (2 µg/ml). The medium was then aspirated and cells were incubated with platelet supernatants (250 µl) previously stimulated with galectins or control medium without platelets supplemented with penicillin/streptomycin and polymyxin (7 µg/ml). Positive and negative controls included Tyrode’s buffer with or without VEGF (20 ng/ml). After 18 h of incubation, MTT was added to each well, and cells were further incubated for 3 h at 37°C. Stop solution was added and aliquots of each well were transferred to a 96-well plate and absorbance at 570 nm was measured. The number of cells was extrapolated from a concentration-cell curve performed for each experiment [30].

### *In vitro* Tube Formation Assay

Prechilled 48-well microtiter plates were coated with growth factor-reduced Matrigel for 2 h at 37°C. HMEC-1 (2.5×10^4^) in Tyrode’s were loaded in each well, preincubated with sucrose (50 mM) or lactose (50 mM) in the presence or absence of a neutralizing anti-VEGFR2 antibody (2 µg/ml), anti-VEGF-A (2 µg/ml), or anti-EGFR (20 µg/ml), for 30 min, and then incubated in the presence of VEGF (20 ng/ml) or EGF (20 ng/ml) (positive controls), or platelet supernatants obtained after stimulation with galectins supplemented with polymyxin (7 µg/ml) for 18 h. Tube formation was analyzed under an inverted light microscope, and the number of branch points in four non-overlapping fields was determined [30].

### Statistical Analysis

The results are expressed as mean ± SEM and were analyzed by one-way analysis of variance followed by Newman-Keuls multiple comparison test to determine significant differences between groups. A *P* value lower than 0.05 was considered statistically significant.

## Results

### Differential Release of VEGF and Endostatin Induced by Gal-1, -3 and -8

**Figure 1:**
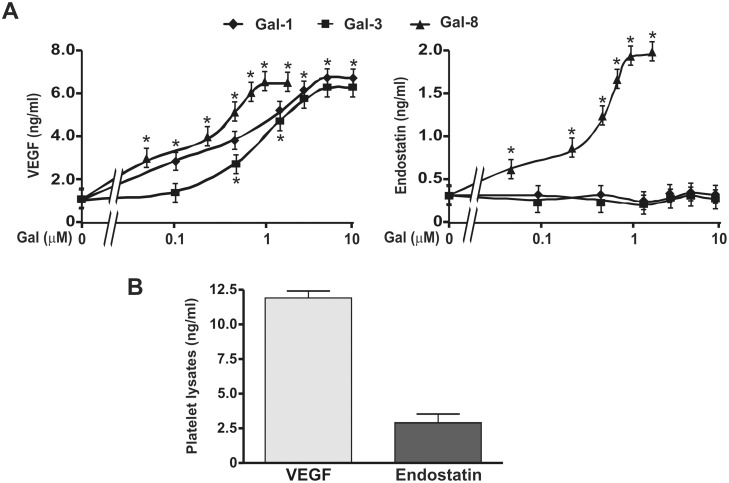
Gal-1, Gal-3 and Gal-8 differentially control the release of VEGF and endostatin by platelets. A) WPs were stimulated with different concentrations of Gal-1, -3 or -8 for 5 min (n = 4; *P<0.05 vs. unstimulated). B) WPs (500 µl at 4×10^8^/ml) were lysed with Triton X100 (0.1%) and sonicated (n = 4). The amounts of VEGF and endostatin in supernatants were quantified by ELISA.

Release of angiogenesis-modulating factors from platelet granules is one of the mechanisms by which platelets can modulate vessel formation [3]. As galectins can stimulate both neovascularization and platelet activation, we examined whether representative members of the three subfamilies of galectins including Gal-1 (‘proto-type’), Gal-3 (‘chimera-repeat’) or Gal-8 (‘tandem-repeat’) can trigger the release of platelet-derived molecules that promote or inhibit angiogenesis such as VEGF or endostatin, respectively. We found that while Gal-8 induced the secretion of VEGF and endostatin, Gal-1 and -3 only promoted the release of VEGF. Of note, even at high concentrations (up to 10 µM), Gal-1 and -3 failed to induce endostatin secretion (Figure 1A). Based on EC_50_ values, we found that Gal-8 induced higher secretion of VEGF (0.25±0.05 µM) than Gal-1 (0.50±0.05 µM) or Gal-3 (1.0±0.3 µM).

The total amount of intraplatelet VEGF and endostatin was measured in platelet lysates (Figure 1B). Platelet activation induced by each galectin led to the release of 50% of the stored VEGF, whereas Gal-8 induced the secretion of 75% of total endostatin.

**Figure 2:**
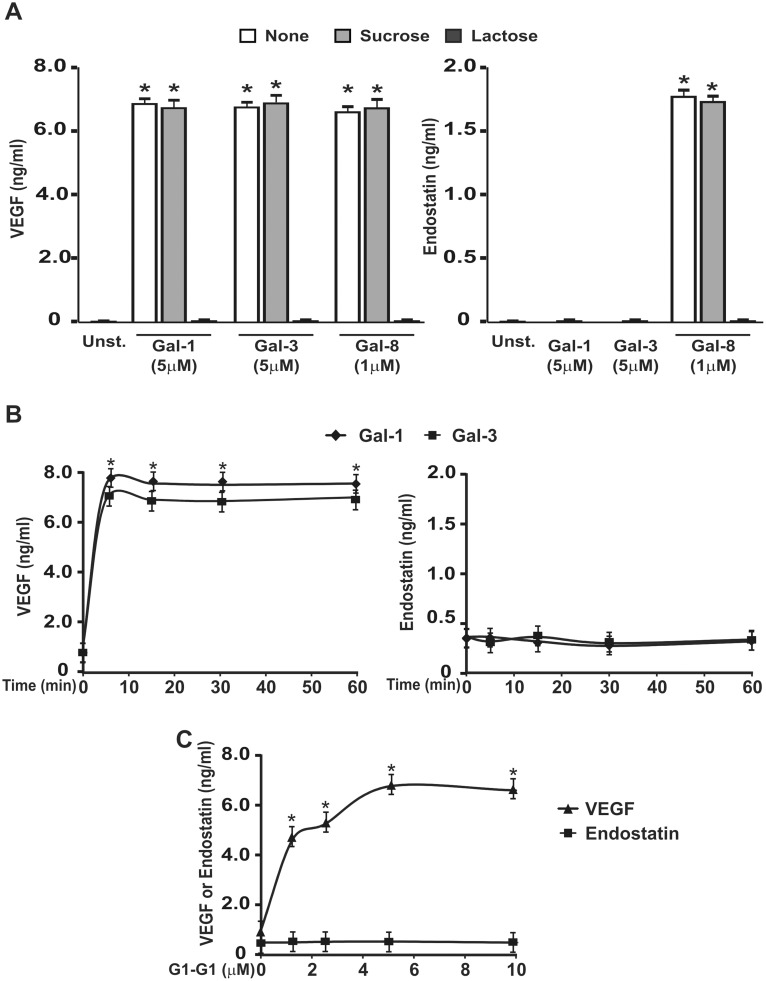
Differential release of VEGF and endostatin is independent of the kinetics of incubation or structural differences among galectins. A) WPs were pre-incubated in the absence (none) or the presence of sucrose (50 mM) or lactose (50 mM) for 30 min and then stimulated with Gal-1 (5 µM), Gal-3 (5 µM), or Gal-8 (1 µM) during 5 min. B) WPs were stimulated with Gal-1 (5 µM) or Gal-3 (5 µM) under stirring conditions for different intervals of time during 60 min. C) WPs were stimulated with different concentrations of a Gal-1 chimera (G1-G1) for 10 min (n = 3; *P<0.05 vs. unstimulated).

The secretion of VEGF induced by each galectin as well as the secretion of endostatin induced by Gal-8 were completely prevented by lactose, a galectin-specific competitor, but not by sucrose, suggesting that these effects involved recognition of glycan residues on the platelet surface (Figure 2A). To examine whether the selective release of VEGF and endostatin induced by Gal-1 and -3 could be associated with differences in the kinetics of platelet stimulation, treatment was extended from 5 to 60 min. Moreover, in order to induce the maximal release of granule cargo, these assays were performed under stirring. Under these conditions, VEGF release was slightly enhanced by galectins; however Gal-1 and Gal-3 still failed to induce endostatin secretion (Figure 2B). A possible explanation for the differential release of endostatin after platelet stimulation with Gal-1, -3 or -8 might rely on the structural differences between the three lectins. To test this hypothesis, the secretion of angiogenesis-related molecules was induced with a protein chimera designed by connecting two sequences of full-length Gal-1 with the linker peptide from Gal-8L isoform, resulting in a combination of the Gal-1 CRD sequence with the Gal-8 dimeric structure. Like Gal-1, the chimeric lectin failed to induce endostatin release suggesting that the dissimilar responses exerted by galectins are not due to their structural composition but probably to the specific CRDs (Figure 2C).

### The Release of VEGF and Endostatin Induced by Galectins is Regulated by different Signaling Pathways

**Figure 3:**
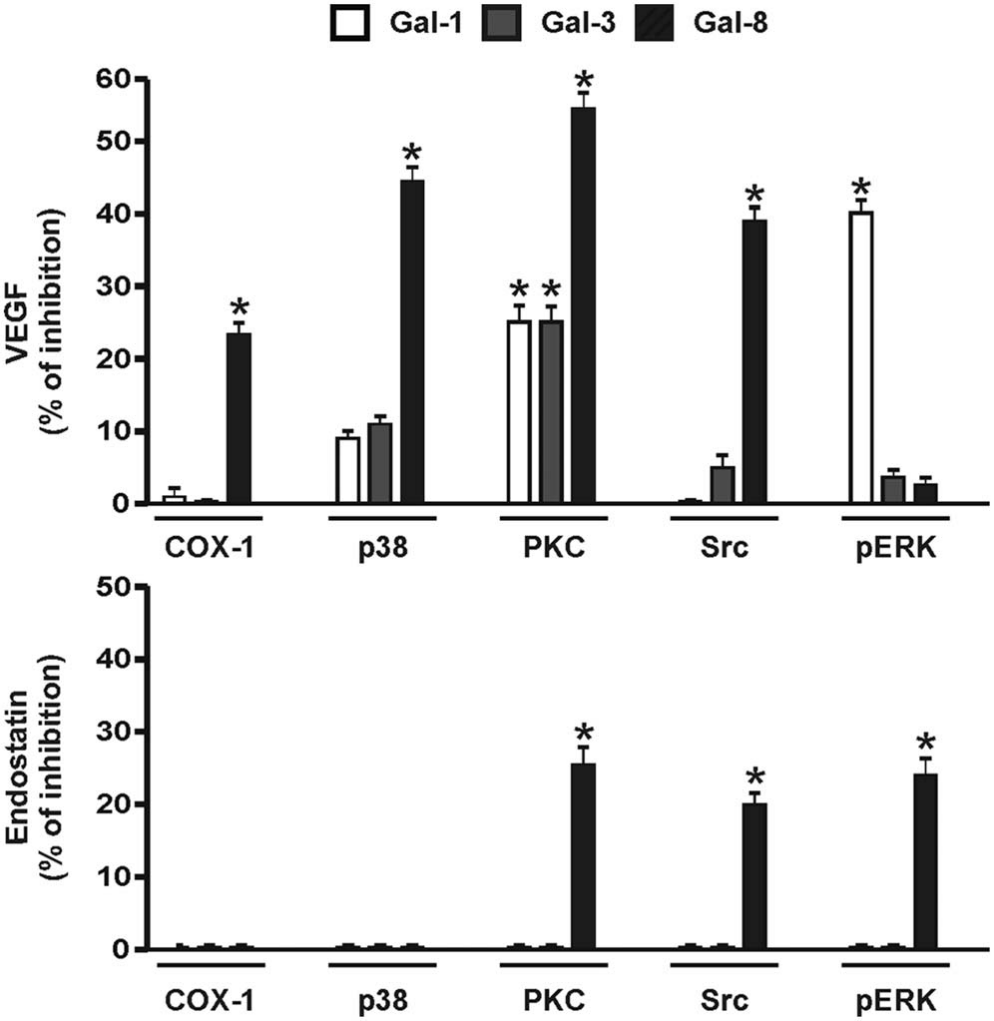
Different signaling pathways mediate the release of VEGF and endostatin induced by individual galectins. WPs were incubated with specific inhibitors of cyclooxygenase-1 (ASA, 500 µM), p38 (SB-203580, 25 µM), PKC (Gö-6983, 1 µM), Src (PP1, 5 µM), or pERK (U-0126, 10 µM) for 30 min. Then, platelets were stimulated with the EC_50_ of Gal-1 (0.5 µM), Gal-3 (1 µM) and Gal-8 (0.25 µM) for 5 min. Results are expressed as percentage of inhibition relative to similar treatment without inhibitor (n = 3; *P<0.05).

To investigate the intracellular signals triggered by galectins to induce VEGF and endostatin release, in the next set of experiments platelet stimulation was performed in the presence of specific inhibitors of the major signaling pathways involved in platelet granule secretion including cyclooxygenase (aspirin), p38 (SB203580), PKC (Gö6983), Src (PP1), and pERK (U0126). To examine possible inhibitory or synergistic effects of these compounds, platelet activation was induced using the EC_50_ of each galectin. Whereas release of VEGF mediated by Gal-8 was partially prevented in platelets that were pretreated with inhibitors of COX-1, PKC, p38 and Src kinases, Gal-1-induced secretion of this proangiogenic factor was inhibited through blockade of PKC and ERK phosphorylation. Moreover, Gal-3-induced VEGF secretion was only prevented upon PKC blockade (Figure 3). Treatment of platelets with aspirin partially inhibited VEGF release triggered by Gal-8 but had no effect on VEGF release induced by Gal-1 or Gal-3 (Figure 3). With regard to endostatin release mediated by Gal-8, it was only slightly decreased in the presence of PKC, Src and ERK inhibitors. Of note, Gal-1 and Gal-3 still failed to induce endostatin secretion in the presence of these inhibitors, excluding the possibility that any of these pathways could interfere with endostatin release.

### Platelet Stimulation by Galectins Promotes Angiogenic Responses

**Figure 4:**
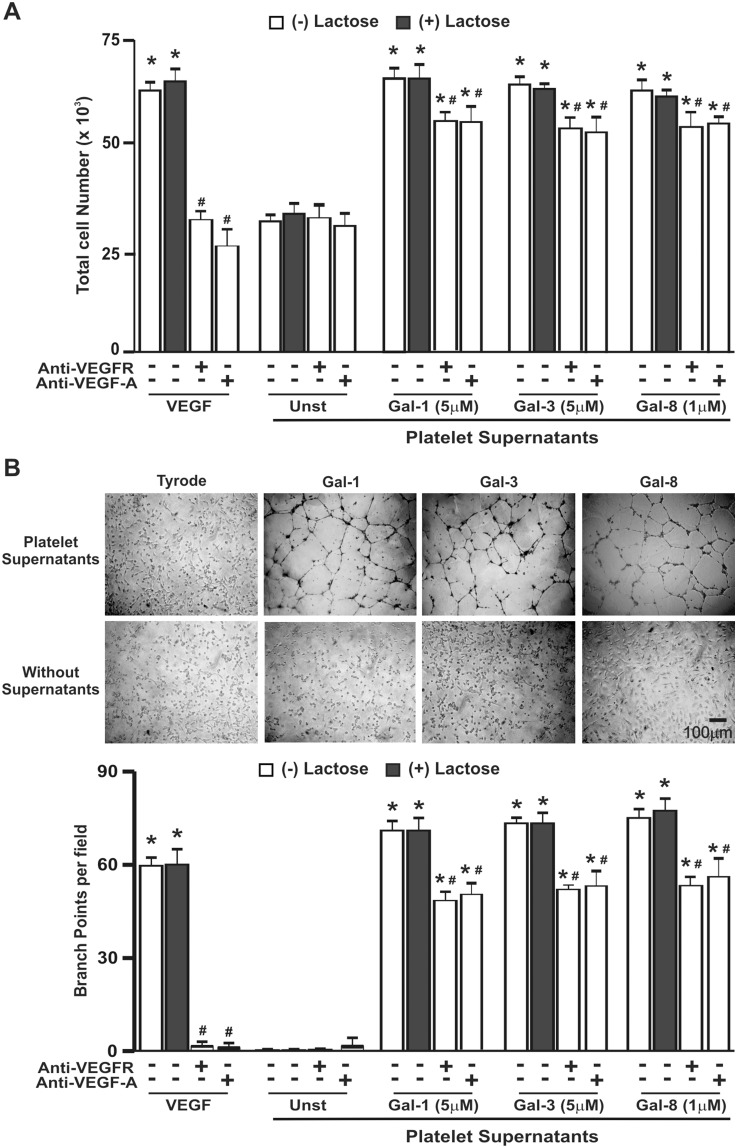
Platelets stimulated by galectins trigger endothelial cell proliferation and capillary-like tube formation. HMEC-1 were preincubated in 48-well plates in the presence of lactose (50 mM), and with anti-VEGFR2, anti-VEGF-A (2 µg/ml), goat or mouse irrelevant IgGs during 30 min. Since proliferation or capillary tube formation were not modify by the different IgGs we only show the values obtained in the absence of antibodies. Then, cells were incubated during 18 h with VEGF (20 ng/ml) (positive control) or with supernatants from platelets that were unstimulated or stimulated with the minimal concentration of galectin to induce maximal VEGF release. A) Endothelial cell proliferation was determined by addition of MTT reagent. Reaction was stopped and the absorbance was determined at 570 nm. B) Tube formation in Matrigel-coated wells was analyzed under an inverted light microscope, and the number of branch points in four non-overlapping fields was determined. The photographs show one individual field of endothelial cells incubated with or without platelet supernatants (n = 4; *P<0.05 vs. unstimulated, #*P*<0.05 vs. without anti-VEGFR2 mAb).

To evaluate the functional impact of galectin effects, we next assessed the angiogenic potential of conditioned medium generated by stimulation of platelets with each individual galectin. Whereas supernatants derived from resting platelets showed no significant effect, supernatants from platelets stimulated with Gal-1,-3 or -8 elicited endothelial cell proliferation and capillary-like tube formation (Figure 4). As previous studies indicated that all three galectins have a direct proangiogenic activity [23], [24], [25], [26], it is conceivable that a variable amount of unbound galectin present in platelet supernatants could be responsible for the observed proangiogenic effect. To rule out this possibility, the assays were repeated in the presence of the galectin competitor lactose. Although lactose effectively blocked the direct stimulatory effect of the three galectins on endothelial cell proliferation and tubule formation (Figure S1), it failed to alter responses triggered by supernatants from galectin-treated platelets, indicating that, under our experimental conditions, the proangiogenic effects were independent of a direct action of these lectins (Figure 4). Altogether, these data demonstrate that although Gal-1, -3 or -8 differ in the pattern of secretion of pro- and antiangiogenic factors, conditioned medium generated after platelet stimulation with any of these three lectins clearly stimulated angiogenic responses. It is well known that platelet-derived VEGF is one of the most abundant molecules in ischemic tissues and plays a leading role in revascularization; however we recently found that platelet-induced angiogenesis triggered by thrombin is independent of VEGF [30]. To test the role of this factor in angiogenesis mediated by galectin-treated platelets, we performed the same experiments in the presence of anti-VEGFR2 or VEGF-A neutralizing antibodies. As shown in Figure 4, cellular proliferation and tubule formation induced by VEGF alone were fully inhibited by these antibodies (96–99% of inhibition). However, when these angiogenic responses were induced by platelets supernatants, only a slight inhibitory effect was observed by either VEGFR2 or VEGF-A blockade (20–25% of inhibition) (Figure 4). Thus, in spite of the considerable increase in VEGF secretion triggered by galectins, the proangiogenic effects triggered by galectin-treated platelets appear to be, at least in part, independent of VEGFR2 signaling.

### Pattern of Pro- and Antiangiogenic Molecules Secreted by Platelets in Response to Gal-1, -3 and -8

**Figure 5:**
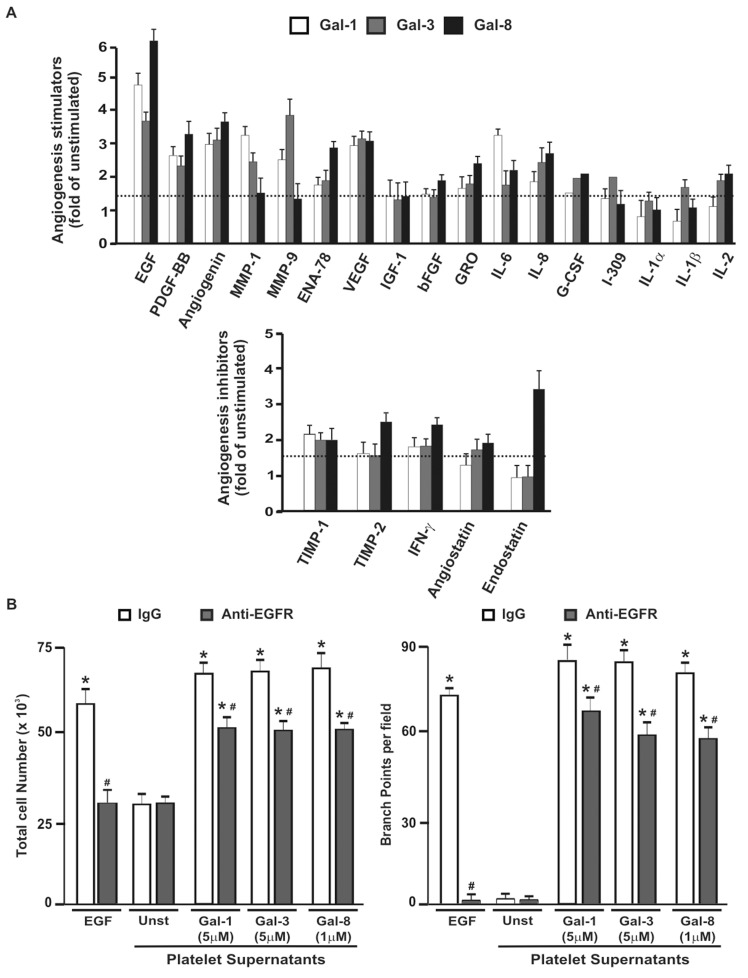
Gal-1, -3 or -8 induce a differential pattern of angiogenic regulators release in platelets. A) WPs were stimulated with Gal-1 (5 µM), Gal-3 (5 µM) or Gal-8 (1 µM) for 5 min. The release of angiogenic factors was determined using a commercial array kit. Release of each molecule induced by individual galectins is expressed as fold increase over unstimulated controls (n = 2). B) HMEC-1 were preincubated with anti-EGFR antibody (20 µg/ml) or irrelevant IgG during 30 min. Then, cells were incubated for 18 h with EGF (20 ng/ml) or with supernatants from unstimulated or galectin-stimulated platelets. Endothelial cell proliferation and tube formation were analyzed (n = 3; *P<0.05 vs. unstimulated, #*P*<0.05 vs. without neutralizing antibody (Ab)).

As angiogenesis induced by supernatants from galectin-treated platelets appears to be mostly VEGF-independent, we then analyzed the pattern of secretion of platelet-derived angiogenic factors triggered by Gal-1, -3 or -8 using an angiogenic microarray kit (Figure 5 and Table S1). Factors increasing at least 1.5 fold were included in the analysis. Remarkably, epidermal growth factor (EGF) was the major factor released upon platelet stimulation by the three galectins**.** The amounts of other factors including PDGF-BB, angiogenin and VEGF, were equivalent in supernatants from platelets treated with either Gal-1, -3 or -8, whereas secretion of other factors was selectively modulated by each individual galectin. While Gal-1 induced maximal release of IL-6 and MMP-1, Gal-3 induced maximal release of I-308, IL-1β and MMP-9 (Figure 5). Moreover, supernatants of platelet stimulated by Gal-8 contained more EGF, ENA-78, bFGF, GRO, TIMP-2, IFN-γ, and endostatin than those from platelets stimulated by Gal-1 or -3 (Figure 5). Altogether these findings suggest that each galectin differentially controls the release of platelet-derived angiogenic factors, which may act in concert to promote angiogenesis.

To further identify the relevant mediator responsible for the proangiogenic activity of platelets, angiogenic responses were induced by platelet supernatants that were previously stimulated with galectins in the presence of an anti-EGF receptor antibody. Similar to VEGF, blockade of EGF activity resulted in moderate inhibition of both endothelial cell proliferation and capillary-like tube formation (Figure 5B). Moreover, simultaneous suppression of VEGF and EGF, did not exert additive or synergic inhibitory effects in platelet-mediated angiogenesis (Figure S2), suggesting that the combined action of other factors accounts for the observed effect.

## Discussion

In this study we provide evidence of an additional mechanism, i.e. the release of platelet-derived angiogenic molecules, by which galectins promote angiogenesis. Although non-structurally related galectins such as Gal-1, -3 and -8 selectively modulated secretion of VEGF and endostatin from platelet α-granules, platelet activation induced by each galectin resulted in a VEGF-independent proangiogenic response, which was likely due to the combined action of several angiogenic mediators.

The ability of all three galectins to trigger VEGF release, but only Gal-8 being able to induce endostatin secretion is in agreement with the new paradigm of platelet α-granule secretion. This new paradigm supports the notion that not all α-granules are morphologically similar, they store different cargo proteins, and they can be selectively activated to release molecules with opposite roles. Recent studies have focused on this novel paradigm in terms of secretion of pro- and antiangiogenic molecules triggered by agonists such as thrombin receptor agonists, ADP or thromboxane A_2_ [5], [7], [8], [9], [10]. Here we demonstrate that the differential release of VEGF or endostatin is not restricted to classical platelet agonists but can also be induced by non-canonical platelet activators such as galectins. Although the structure and dynamics of α-granule secretion is still under debate, different hypotheses seem to converge in the notion that selective signaling pathways might be involved in the regulation of α-granule content exocytosis. In this sense, differences in the release of pro- and antiangiogenic molecules induced by galectins were not associated with differences in the kinetics of secretion or to structural divergences, but rather with distinct signaling pathways triggered by each galectin. While Gal-1 triggered PKC and ERK activation to induce VEGF release, Gal-3 activated a PKC-dependent pathway to stimulate the same biological response. On the other hand, release of VEGF by Gal-8 required not only the activation of COX-1 but also activation of PKC, p38, and Src kinases.

Interestingly, conditioned media derived from platelets activated by each galectin all elicited endothelial cell proliferation and capillary-tube formation, two cellular events linked to angiogenesis. These data indicate that regardless of the differential effect of Gal-1, -3 or -8 on the secretion of angiogenic factors, galectin-induced platelet activation results in promotion of an angiogenic phenotype.

In contrast to the traditional role of VEGF in platelet-mediated angiogenesis, we found that suppressing VEGF signaling using anti-VEGFR2 or anti-VEGF-A antibodies, did not considerably alter endothelial cell responses, suggesting that this factor could be dispensable for the angiogenic activity of platelets when activated in response to galectin stimulation. Interestingly, we found that blockade of EGF, the mayor angiogenic factor released from platelets upon stimulation with galectins partially inhibited angiogenesis mediated by supernatants from galectin-stimulated platelets. Moreover, a similar degree of inhibition was observed when the activity of both VEGF-A and EGF was inhibited. These results suggest that the angiogenic activity mediated by platelets is a complex process that appears to be due to the combined activity of several angiogenesis-related molecules released from galectin-stimulated platelets. In this regard, we recently demonstrated that platelet-induced angiogenesis by thrombin also involves the activity of several angiogenic modulators but not VEGF [30]. Altogether, these studies suggest that platelets can release pro- and antiangiogenic substances and different agonists can selectively regulate this process. Moreover, it could be speculated that in the activated normal or tumor endothelium galectins are differentially regulated [31], [32], [33] and contribute to tumor vascularization by stimulating the release of diverse proangiogenic mediators. Interestingly, the net biological effect appears to be proangiogenic regardless of the platelet agonist used. This implies that the proangiogenic effect of galectins via platelets contributes to a positive feedback that influences tumor progression. However, we do not rule out the possibility that, in an alternative pathologic setting the contribution of platelet-derived antiangiogenic mediators might gain relevance. In this sense, it has been demonstrated that concentration of angiogenesis regulatory proteins in platelets, although relatively constant and stable under physiologic conditions, is substantially modified by the presence of a growing tumor. Specifically it has been shown that changes in major platelet antiangiogenic factors such as platelet-associated PF-4 [34] and TSP-1 [35] are upregulated in platelets of tumor-bearing mice. Regarding TSP-1, this phenomenon has been found to be a consequence of both, increased levels of TSP-1 mRNA in megakaryocytes, as well as increased numbers of these cells in the bone marrow of tumor-bearing mice [35]. Thus, it has been suggested that the production and delivery of antiangiogenic molecules by platelets might serve as a critical early host response to suppress vascular supply during tumor growth, emphasizing the potential value of these molecules as indicators for tumor growth and regression. Since the early antiangiogenic response triggered by platelets could be overcome during tumor progression inducing a switch toward a platelet proangiogenic phenotype, more data are needed to establish whether the platelet proteome changes after longer periods following the establishment of a tumor. Supporting these findings, it has been recently demonstrated that the tumor cell line MCF-7 and the platelet agonist ADP, induce the release of VEGF but not endostatin from platelets, thus promoting migration and tubulogenesis in endothelial cells [9]. In addition, conditioned medium from MCF-7-stimulated platelets contain a greater number of proangiogenic factors compared to conditioned medium from ADP-stimulated platelets suggesting that the process of platelet activation with MCF-7 cells involves activation of additional mechanisms rather than just ADP [9]. As MCF-7 cells express several members of the galectin family, including Gal-1, -3 and -8 [36], and GPIIb/IIIa and GPIb are essential for transducing galectin signaling [20], [22], platelet activation mediated by galectins could have evolved as a mechanism complementary to ADP to promote a tumor proangiogenic phenotype.

In summary, our data highlight an additional mechanism, based on the secretion of platelet-derived angiogenic factors, by which galectins contribute to angiogenesis. As galectins are up-regulated in endothelial cells and tumors in angiogenic-related disorders, and galectin stimulation activates platelets to secrete soluble proangiogenic factors, selective targeting of these pathways may contribute to delineate novel therapeutic approaches in order to promote tissue regeneration and attenuate atherosclerosis, ischemic ulcers, diabetes and cancer.

## Supporting Information

Figure S1 **Lactose inhibits endothelial cell proliferation and capillary-like tube formation induced by galectins.** A) HMEC-1 (2.5×10^4^/well) were preincubated in 48-well plates in the presence of lactose (50 mM) for 30 min. Then, cells were incubated in buffer Tyrode’s with or without VEGF-A (20 ng/ml) (positive control) or with Gal-1, -3 or -8 at the indicated concentrations. After 18 h of incubation, MTT reagent was added to each well. Reaction was stopped and the absorbance was determined at 570 nm (n = 3; *P<0.05 vs. unstimulated). B) HMEC-1 (2.5×10^4^) were preincubated in Matrigel-coated wells in the presence of lactose for 30 min. Then, cells were incubated with VEGF-A (positive control) or with Gal-1, -3 or -8 at the indicated concentrations for 18 h. Tube formation was analyzed under an inverted light microscope and the number of branch points was determined in four non-overlapping fields (n = 3; *P<0.05 vs. unstimulated).
(TIF)

Figure S2 **Roles of VEGF and EGF in angiogenesis induced by galectin-stimulated platelets.** HMEC-1 were preincubated in 48-well plates with anti-VEGF-A (2 µg/ml), anti-EGFR (20 µg/ml), or irrelevant mouse and rabbit IgG controls during 30 min. Since proliferation or capillary tube formation were not modify by the different IgGs we only show the values obtained in the absence of antibodies (none). Then, cells were incubated during 18 h with VEGF (20 ng/ml) and EGF (20 ng/ml) (positive controls), or with supernatants from unstimulated or galectin-stimulated platelets. A) Endothelial cell proliferation was determined by addition of MTT reagent. B) Tube formation in Matrigel-coated wells was analyzed under an inverted light microscope, and the number of branch points in four non-overlapping fields was determined. The photographs show one individual field of endothelial cells incubated with supernatants from Gal-1-stimulated platelets (n = 3; *P<0.05 vs. unstimulated, #*P*<0.05 vs. without neutralizing antibodies (Abs)).
(TIF)

Table S1 **Platelet-derived angiogenesis modulators measured by the Antibody Array G Series 1000 from Raybiotech (n = 2).**
(DOC)
