# Supplementary figures and images for: Control of Angiogenesis by Galectins Involves the Release of Platelet-Derived Proangiogenic Factors
Source: PLoS One. 2014 Apr 30;9(4):e96402. doi: 10.1371/journal.pone.0096402 (PMC4005776; doi:10.1371/journal.pone.0096402)

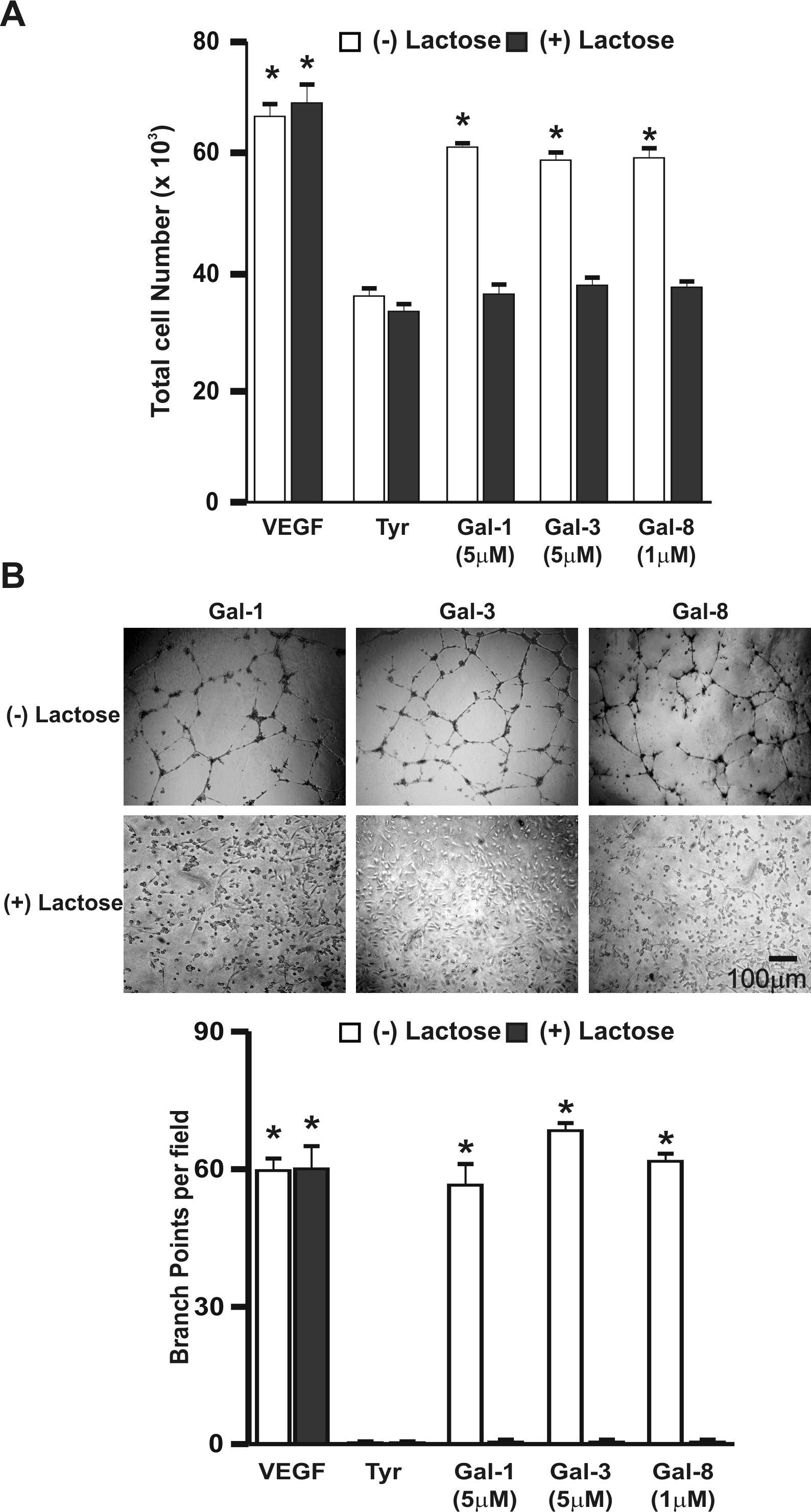

Supplement: Figure S1 — Lactose inhibits endothelial cell proliferation and capillary-like tube formation induced by galectins. A) HMEC-1 (2.5×10^4^/well) were preincubated in 48-well plates in the presence of lactose (50 mM) for 30 min. Then, cells were incubated in buffer Tyrode’s with or without VEGF-A (20 ng/ml) (positive control) or with Gal-1, -3 or -8 at the indicated concentrations. After 18 h of incubation, MTT reagent was added to each well. Reaction was stopped and the absorbance was determined at 570 nm (n = 3; *P<0.05 vs. unstimulated). B) HMEC-1 (2.5×10^4^) were preincubated in Matrigel-coated wells in the presence of lactose for 30 min. Then, cells were incubated with VEGF-A (positive control) or with Gal-1, -3 or -8 at the indicated concentrations for 18 h. Tube formation was analyzed under an inverted light microscope and the number of branch points was determined in four non-overlapping fields (n = 3; *P<0.05 vs. unstimulated). (TIF) [file pone.0096402.s001.tif]
