# Supplementary material for: Control of Angiogenesis by Galectins Involves the Release of Platelet-Derived Proangiogenic Factors
Source: PLoS One. 2014 Apr 30;9(4):e96402. doi: 10.1371/journal.pone.0096402 (PMC4005776; doi:10.1371/journal.pone.0096402)
Supplement: Table S1 — Platelet-derived angiogenesis modulators measured by the Antibody Array G Series 1000 from Raybiotech (n = 2). (DOC) [file pone.0096402.s003.doc]

**Table S1. Platelet-derived angiogenesis modulators measured by the Antibody Array G Series 1000 from Raybiotech (n=2).**

|  | ***Platelet-derived angiogenesis Stimulators*** | | | | | | |
| --- | --- | --- | --- | --- | --- | --- | --- |
|  | Unstimulated | Gal-1  (5 µM) | Gal-3  (5 µM) | Gal-8  (1 µM) | Gal-1  (5 µM) | Gal-3  (5 µM) | Gal-8  (1 µM) |
|  | mean of fluorescence | | | | fold of unstimulated | | |
| RANTES | 21087 | 25065 | 29199 | 24098 | 1,2 | 1,4 | 1,1 |
| Angiopoietin-1 | 10443 | 12085 | 11891 | 14010 | 1,2 | 1,1 | 1,3 |
| Angiopoietin-2 | 2290 | 1962 | 1448 | 2521 | 0,9 | 0,6 | 1,1 |
| PDGF-BB | 5151 | 13882 | 12310 | 17223 | 2,7 | 2,4 | 3,3 |
| Angiogenin | 4225 | 12864 | 13428 | 15772 | 3,0 | 3,2 | 3,7 |
| EGF | 1129 | 5470 | 4211 | 7000 | 4,8 | 3,7 | 6,2 |
| ENA-78 | 890 | 1604 | 1707 | 2611 | 1,8 | 1,9 | 2,9 |
| VEGF | 202 | 605 | 650 | 638 | 3,0 | 3,2 | 3,2 |
| IGF-1 | 266 | 388 | 361 | 382 | 1,5 | 1,4 | 1,4 |
| b FGF | 187 | 286 | 267 | 365 | 1,5 | 1,4 | 1,9 |
| GRO | 663 | 1113 | 1215 | 1635 | 1,7 | 1,8 | 2,5 |
| IL-6 | 216 | 720 | 391 | 485 | 3,3 | 1,8 | 2,2 |
| IL-8 | 373 | 706 | 929 | 1034 | 1,9 | 2,5 | 2,8 |
| G-CSF | 270 | 423 | 538 | 575 | 1,6 | 2,0 | 2,1 |
| I-309 | 438 | 604 | 897 | 530 | 1,4 | 2,0 | 1,2 |
| IL-1 alpha | 300 | 251 | 389 | 308 | 0,8 | 1,3 | 1,0 |
| IL-1 beta | 326 | 225 | 560 | 359 | 0,7 | 1,7 | 1,1 |
| IL-2 | 401 | 461 | 775 | 856 | 1,1 | 1,9 | 2,1 |
| MMP-1 | 327 | 1084 | 777 | 531 | 3,3 | 2,4 | 1,6 |
| MMP-9 | 326 | 855 | 1280 | 424 | 2,6 | 3,9 | 1,3 |
| LEPTIN | n.d. | | | | | | |
| TGF-*beta*1 | n.d. | | | | | | |
| TPO | n.d. | | | | | | |
| VEGF-D | n.d. | | | | | | |
| GM-CSF | n.d. | | | | | | |
| IL-10 | n.d. | | | | | | |
| MCP-1 | n.d. | | | | | | |
| MCP-3 | n.d. | | | | | | |
| MCP-4 | n.d. | | | | | | |
| PECAM-1 | n.d. | | | | | | |
| Tie-2 | n.d. | | | | | | |
| TNF-α | n.d. | | | | | | |
| uPAR | n.d. | | | | | | |
| VEGFR2 | n.d. | | | | | | |
| VEGFR3 | n.d. | | | | | | |
|  | ***Platelet-derived angiogenesis inhibitors*** | | | | | | |
|  | Unstimulated | Gal-1  (5 µM) | Gal-3  (5 µM) | Gal-8  (1 µM) | Gal-1  (5 µM) | Gal-3  (5 µM) | Gal-8  (1 µM) |
|  | mean of fluorescence | | | | fold of unstimulated | | |
| TIMP-1 | 2176 | 4905 | 4350 | 4367 | 2,3 | 2,0 | 2,0 |
| TIMP-2 | 2822 | 4402 | 4239 | 6892 | 1,6 | 1,5 | 2,4 |
| IFN-γ | 280 | 493 | 501 | 660 | 1,8 | 1,8 | 2,4 |
| Angiostatin | 434 | 544 | 731 | 812 | 1,3 | 1,7 | 1,9 |
| Endostatin | 165 | 148 | 152 | 552 | 0,9 | 0,9 | 3,3 |
| IL-4 | n.d. | | | | | | |
| I-TAC | n.d. | | | | | | |
| PIGF | n.d. | | | | | | |

bFGF, basic fibroblast growth factor; EGF, epidermal growth factor; ENA-78, epithelial neutrophil-activating peptide; G-CSF, granulocyte-colony stimulating factor; GM-CSF, granulocyte macrophage-colony stimulating factor; IGF-1, insulin-like growth factor 1; IL, interleukin; IFNγ, interferon-gamma; I-309 (CCL1), small inducible cytokine A1; GRO, growth-regulated oncogene; MMP, metalloprotease; n.d, not detected; PDGF-BB, platelet-derived growth factor-BB; PECAM-1, platelet endothelial cell adhesion molecule-1; PIGF, placenta growth factor-1; RANTES (CCL5), regulated and normal T cell expressed and secreted; TGF-*beta*1, transforming growth factor 1 beta; TPO, thrombopoietin; Tie-2, tyrosine-protein kinase receptor; TIMP, tissue inhibitor of metalloprotease; TNF-α, tumor necrosis factor-alpha; VEGF, vascular endothelial growth factor; VEGFR, vascular endothelial growth factor receptor; uPAR, urokinase-type plasminogen activator receptor.
